# Peri-ictal activation of dorsomedial dorsal raphe serotonin neurons reduces mortality associated with maximal electroshock seizures

**DOI:** 10.1093/braincomms/fcae052

**Published:** 2024-03-14

**Authors:** Alexandra N Petrucci, Allysa R Jones, Benjamin L Kreitlow, Gordon F Buchanan

**Affiliations:** Interdisciplinary Graduate Program in Neuroscience, Carver College of Medicine, University of Iowa, Iowa City, IA 52242, USA; Department of Neurology, Carver College of Medicine, University of Iowa, Iowa City, IA 52242, USA; Iowa Neuroscience Institute, Carver College of Medicine, University of Iowa, Iowa City, IA 52242, USA; Department of Neurology, Carver College of Medicine, University of Iowa, Iowa City, IA 52242, USA; Iowa Neuroscience Institute, Carver College of Medicine, University of Iowa, Iowa City, IA 52242, USA; Interdisciplinary Graduate Program in Neuroscience, Carver College of Medicine, University of Iowa, Iowa City, IA 52242, USA; Department of Neurology, Carver College of Medicine, University of Iowa, Iowa City, IA 52242, USA; Iowa Neuroscience Institute, Carver College of Medicine, University of Iowa, Iowa City, IA 52242, USA; Department of Neurology, Carver College of Medicine, University of Iowa, Iowa City, IA 52242, USA; Iowa Neuroscience Institute, Carver College of Medicine, University of Iowa, Iowa City, IA 52242, USA

**Keywords:** SUDEP, serotonin, dorsal raphe nucleus, dorsomedial dorsal raphe nucleus

## Abstract

Over one-third of patients with epilepsy will develop refractory epilepsy and continue to experience seizures despite medical treatment. These patients are at the greatest risk for sudden unexpected death in epilepsy. The precise mechanisms underlying sudden unexpected death in epilepsy are unknown, but cardiorespiratory dysfunction and arousal impairment have been implicated. Substantial circumstantial evidence suggests serotonin is relevant to sudden unexpected death in epilepsy as it modulates sleep/wake regulation, breathing and arousal. The dorsal raphe nucleus is a major serotonergic center and a component of the ascending arousal system. Seizures disrupt the firing of dorsal raphe neurons, which may contribute to reduced responsiveness. However, the relevance of the dorsal raphe nucleus and its subnuclei to sudden unexpected death in epilepsy remains unclear. The dorsomedial dorsal raphe may be a salient target due to its role in stress and its connections with structures implicated in sudden unexpected death in epilepsy. We hypothesized that optogenetic activation of dorsomedial dorsal raphe serotonin neurons in *TPH2-ChR2-YFP* (*n* = 26) mice and wild-type (*n* = 27) littermates before induction of a maximal electroshock seizure would reduce mortality. In this study, pre-seizure activation of dorsal raphe nucleus serotonin neurons reduced mortality in *TPH2-ChR2-YFP* mice with implants aimed at the dorsomedial dorsal raphe. These results implicate the dorsomedial dorsal raphe in this novel circuit influencing seizure-induced mortality. It is our hope that these results and future experiments will define circuit mechanisms that could ultimately reduce sudden unexpected death in epilepsy.

## Teaser

Petrucci *et al*. report that pre-seizure optogenetic stimulation of serotonergic neurons in the dorsomedial dorsal raphe reduces mortality in the maximal electroshock seizure model without affecting seizure duration or severity. Further research into the networks underlying this effect will increase our understanding of preventing death in epilepsy.

## Graphical Abstract

**Graphical Abstract:**
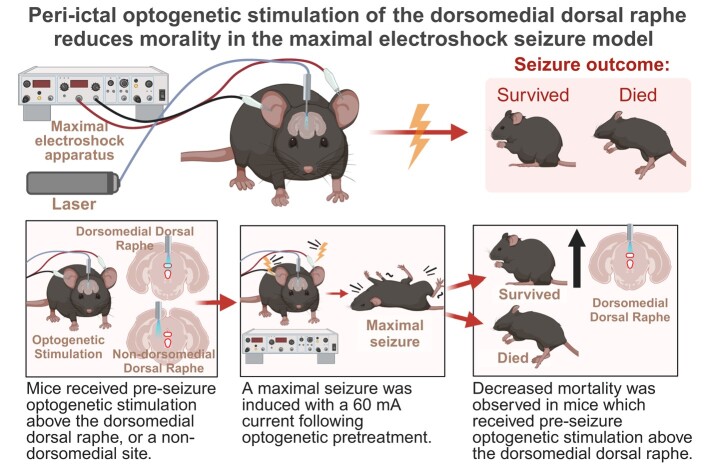

## Introduction

Epilepsy is an enormous public health burden affecting over 65 million individuals worldwide.^1^ Among those diagnosed with epilepsy, about one-third will develop refractory epilepsy and continue to experience seizures despite medical treatment.^2^ This patient population is at greatest risk for sudden unexpected death in epilepsy (SUDEP).^3^ SUDEP accounts for over 100 000 years of potential life lost to neurological disease annually.^4^ Despite this, the precise pathophysiology of SUDEP is unknown and there are no existing therapies to prevent SUDEP.

Cardiorespiratory failure and impairment of arousal are implicated in SUDEP.^3,5^ Seizures can provoke apnoeas,^6^ which cause oxygen desaturation^7^ and increase end-tidal CO_2_.^8^ Tachycardia,^9^ bradycardia^10^ and cardiac arrythmias^10^ are also provoked by seizures. In nine SUDEP events recorded in epilepsy monitoring units, terminal apnoea is preceded by terminal asystole.^11^ Therefore, much SUDEP research is directed towards understanding seizure-induced respiratory dysfunction. Seizures may also interfere with the firing of arousal-promoting networks such as the dorsal raphe nucleus (DRN)^12^ and reduce consciousness.^13^ Most cases of SUDEP occur in a prone position,^14^ thus an inability to arouse to stimuli such as CO_2_ consequent to ictal disruption of arousal networks could increase SUDEP risk.^15^ However, the role of the DRN in seizure-induced mortality remains unknown.

The unpredictable and often unwitnessed nature of SUDEP presents a challenge for extrapolating mechanisms, biomarkers or potential treatments from patient data. Thus, animal models of seizures and epilepsy are crucial to understanding the aetiology of SUDEP. The use of electrically induced seizures to test drug efficacy dates to at least 1937 with Putnam and Merrit’s seminal work testing the anticonvulsant properties of phenytoin.^16^ In the maximal electroshock seizure (MES) model, a single, maximal seizure is induced in a seizure-naive animal either using incremental current increases—MES thresholding^17^—or a suprathreshold current passed transauricularly or transcorneally.^17,18^ MES protocols have been utilized extensively in antiepileptic drug research,^19^ and more recently have been utilized to study potential mechanisms underlying SUDEP and seizure-induced mortality.^20-24^ There are several advantages to studying seizure-induced mortality in the MES model. It is an inducible model with a high mortality rate,^25^ facilitating research into seizure-induced death. Controlled seizure induction facilitates the use of pharmacological or neuromodulatory pretreatments. MES also recapitulates known facets of SUDEP since respiratory cessation is implicated in MES seizure-induced death.^21^

We hypothesized that pre-seizure activation of DRN serotonin (5-HT) neurons would decrease mortality in the MES model. The DRN can be divided into subregions such as dorsomedial (DRD), ventral (DRV) and ventrolateral (DRVL) which have diverse anatomical connections and roles in behaviour.^26^ Thus, it is possible that DRN subregions have varying effects on mortality. Here, we focused on the DRD due to its role in stress/arousal^27^ and its interconnectivity with other structures implicated in SUDEP, such as the basolateral and central amygdala^28,29^ and the bed nucleus of the stria terminalis (BNST).^30^ This hypothesis was tested by activating DRD 5-HT neurons using optogenetics in a serotonin neuron-specific line (*TPH2-ChR2-YFP*) and wild-type (WT) littermates before induction of a maximal seizure. The effect of DRN subnuclei on seizure-induced mortality, seizure duration and seizure severity is unknown. We found that survival was enhanced in mice with implants overlying the dorsomedial subregion of the DRN, the DRD. Here, we further elucidated the anatomical connections of the DRD and propose a putative network that may reduce risk of seizure-induced mortality in MES.

## Materials and methods

### Animals

Male and female *TPH2-ChR2-YFP* mice and WT littermates between 8 and 28 weeks were utilized for MES experiments. *TPH2-ChR2-YFP* mice express the excitatory non-specific cation channel channelrhodopsin2 (ChR2) exclusively in serotonergic, tryptophan hydroxylase 2 (TPH2) expressing cells. This allows for selective activation of 5-HT neurons via optogenetics. This strain is maintained by breeding mice hemizygous for *TPH2-ChR2-YFP* to WT littermates. The WT mice do not express ChR2 and are used as controls in the experiments. The original stock was obtained from Jackson Labs (Bar Harbor, ME, USA) and has since been backcrossed to C57Bl/6J at the animal husbandry facilities at the University of Iowa. Retrograde staining was performed using WT littermates from *Pet1*-Cre mice within our colony. These mice were originally obtained from Jackson Labs (B6.Cg-Tg[Fev-cre]1Esd/J) and backcrossed to C57Bl/6J. Mice within each genotype were randomly assigned to cohorts. Mice were selected for MES in a random order on trial days. Experimenters were kept blinded to genotype following surgical procedures until data analysis was completed.

The experimental procedures were conducted in compliance with the Institutional Animal Care and Use Committee at the University of Iowa Carver College of Medicine. Mice were housed in a 12:12 light:dark cycle (6:00 AM to 6:00 PM) with food and water available ad libitum. Health checks were performed daily. Throughout procedures, animal pain and distress were minimized. All experiments were performed 4–9 h after the start of the light phase. The estrus state of female animals was assessed prior to MES with vaginal cytology.^31^

### Surgical procedures

#### General

Mice were anaesthetized with isoflurane (2–5% induction; 0.5–2% maintenance) for implantation of EEG and EMG electrodes, and an optic fibre. Body temperature was regulated with a heating pad (Physiosuite; Kent Scientific, Torrington, CT, USA). Meloxicam (2.0 mg/kg, i.p.) was administered preoperatively, immediately postoperatively and at 24 and 48 h for pain management. The animal’s scalp was shaved, and their head was secured in a stereotax (51730; Stoelting Co., Wood Dale, IL, USA). Antibiotic ophthalmic ointment (24208-780-55; Bausch and Lomb, Saint Louis, MO, USA) was applied to their eyes, and their scalp was prepared with betadine and 70% ethanol. The depth of anaesthesia was monitored by toe pinch every 15 min. Electrolyte solution (i.p., 0.3 mL; 00336; Abbott Nutritional Products, Abbott Park, IL, USA) was administered as needed for rehydration. Thick cyanoacrylate adhesive (PT-33; Pacer Technology—ZAP, Ontario, CA, USA) and dental acrylic (Lang Dental, Wheeling, IL, USA) were applied to stabilize the implants and reduce noise. Animals recovered in home cages for at least 1 week following surgery.

#### EEG/EMG and optic fibre implantation

Small screws (1.0 mm thread, 4.1 mm length, 0.8 mm shaft diameter; #80404; Vigor Optical, Carlstadt, NJ, USA) soldered to stainless steel wires were threaded inside burr holes drilled into the skull (two 2 mm ± 2 mm anterior to bregma, two 2 mm ± 2 mm anterior to lambda, ∼1.5 mm lateral to midline) to act as EEG electrodes. An additional hole was created above the DRN (AP: −4.6, ML: 0, DV: −2.90 in mm from bregma) for the optic fibre (8 mm, 240 µm diameter, 0.22 NA; MFC_200/240-0.22_4mm_ZF1.25_FLT; Doric Lenses; Quebec, Canada). After the optic fibre was inserted and stabilized, the wires on the EEG electrodes were twisted and soldered to wires emerging from a six-pin socket (853-41-006-30-001000; Millmax Co., Oyster Bay, NY, USA). Two wires acted as EMG electrodes and were inserted bilaterally into the nuchal muscles. The skin surrounding the headcap was closed with 5-0 nylon suture (668G; Ethilon, Inc., Somerville, NJ, USA).

### Experimental procedures

#### Maximal electroshock procedure

At least 1 week after surgical recovery, all mice were habituated to the MES procedure. The animals were placed into a recording chamber and acclimated to the toothless, stainless steel alligator clips for at least 1 h/day on 2 consecutive days. Saline-moistened gauze coated the alligator clips to increase conductivity and protect the animal’s ears. Trials were conducted the day following the last habituation. Ear clips, an EEG pre-amplifier and an optical fibre patch cord (MFC_200/240/900-0.22_0.6m_FC-ZF1.25[F]; Doric Lenses) were then attached to the mouse. Plethysmography data was collected to visualize breathing and identify the onset of apnoea as described below.

After an additional 15 min habituation period, the banana plugs attached to the ear clips were plugged into the Rodent Shocker pulse generator (D-79232; Harvard Apparatus) for a pre-trial 0 mA conductivity test. Then, a single 50 mA (0.2 s, 60 Hz sine wave) stimulus was administered to induce a maximal seizure.^21,23^ All seizures were induced during wakefulness. Video and EEG were utilized to determine mortality, seizure duration and extension-to-flexion (*E*/*F*) ratios. Higher *E*/*F* ratios are a surrogate marker of seizure severity. *E*/*F* ratios were calculated by dividing the time the mouse spent with hind limb extension (≥90°) by hind limb flexion (≤90°).^32^ Video data were unable to be analysed from two WT and two *TPH2-ChR2-YFP* mice.

#### Optogenetics

On the day of the MES procedure, optical light power (∼12.5 mW to account for 25% loss) was tested with a digital optical power metre (P0014083; Thor Labs, Dachau, Germany) prior to set-up. After the 0 mA conductivity test and collection of baseline plethysmography data, all mice (both the *TPH2-ChR2-YFP* and WT mice) received light stimulation (473 nm, 4 Hz, 10 mW, 5 ms; Opto Engine, LLC., Midvale, UT, USA) was applied for 300 s pre-ictally and persisted until first post-ictal breath or death. DRN 5-HT neurons fire slowly (1–4 Hz)^33^; thus, the 4 Hz laser frequency was chosen to complement the native firing rate. The 5-HT neurons lose fidelity to optogenetic stimulation above 10 Hz.^34^

#### Whole-body plethysmography

On the day of experiments, the animal’s temperature was determined via a rectal probe connected to a digital thermometer (401014; ExTech Instruments, Nashua, NH, USA). Air temperature (∼20–22°C) was also monitored throughout the experiments. The chamber used for the maximal electroshock seizure experiences is outfitted for plethysmography. Thus, mice were habituated to these chambers as described previously. The EEG pre-amplifier cable and optic patch cords were routed through a hole in the lid. To render the chamber airtight, thumb screws were tightened to join the lid and box and vinyl-coated laboratory tape (89097; VWR International, Radnor, PA, USA) was applied to seal the cable exit point. Room air (21% O_2_, 79% N_2_; Praxair Inc., Cedar Rapids, IA, USA) flowed into the chamber at 0.40–0.45 L/min through a luer-lock port. Airflow was monitored via a flow metre (32907-67; Cole-Parmer, Vernon Hills, IL, USA), and a vacuum regulator (V-800-30; Airtrol Components Inc., New Berlin, WI, USA) was utilized to produce steady-state pressure within the chamber.

The recording chamber was outfitted with an ultralow pressure transducer (DC002NDR5; Honeywell International, Minneapolis, MN, USA) attached to an analogue-to-digital converter (PCI-6221; National Instruments, Austin, TX, USA) that transmitted data to a desktop computer. Each breath was then plotted in real-time using a custom MATLAB script. Following the 15 min habituation period, calibration was conducted by delivering metered breaths (300 μL, 150/min; MiniVent 845; Hugo Sachs Elektronik, Grünstraße, Germany) to the chamber for 100 s. Baseline breathing was then collected for an additional 100 s. Breathing data continued to be collected during the optogenetic stimulation and continued until experiment termination (animal death, or 300 s following survival). Plethysmography was initially collected to identify the onset of terminal apnoea. A subset of animals was analysed for changes in respiratory parameters during the 300 s optogenetic stimulation.

#### Retrograde tracing

Sites of interest were chosen for retrograde tracing based on prior literature searches: BNST (AP: −0.30, ML: −0.88, DV: −4.0, in mm from bregma), pedunculopontine tegmental nucleus (AP: −4.5, ML: −1.13, DV: −3.5), basolateral amygdala (AP: −1.3, ML: −2.8, DV: −2.68) and hippocampus (HPC, AP: −1.75, ML: −0.75, DV: −2.00). *Pet1*-WT mice were prepared for surgery and received injections of the fluorescent dye Fluorogold (FG; 10 nL; 0.25–5% in ddH_2_O; Fluorochrome, Denver, CO, USA) into a target site over 2 min using a Hamilton syringe (6545901; Hamilton Company, Reno, NV, USA). The syringe remained stationary for 4–5 min and then was slowly removed to minimize diffusion along the tract. Animals were returned to their home cages for 3 days before being sacrificed to ensure the FG was retrogradely transported to the somas.

### Data collection and analysis

#### EEG/EMG data acquisition

EEG/EMG data were collected via a preamplifier (10×; 8202-SL; Pinnacle Technology, Lawrence, KS, USA) linked to a commutator (8204; Pinnacle Technology) and a digital conditioning amplifier (Model 440 Instrumentation Amplifier; Brownlee Precision; San Jose, CA, USA). The EEG signals were further amplified 50 000× and band-pass filtered from 0.3 to 300 Hz. The EMG signals were amplified 50 000× and band-pass filtered from 10 to 1000 Hz. Data were digitized with an analogue-to-digital converter (1000 Hz; PCI-6221 or NI-USB-6009; National Instruments) and compiled using custom MATLAB scripts.

#### Plethysmography analysis

Plethysmography signal was amplified 100×, band-pass filtered (0.3–30 Hz) and digitized via an analogue-to-digital converter. Frequency (fR), tidal volume (VT) and minute ventilation (VE) were determined from artifact-free epochs that precluded sniffing and any movement. fR was calculated by identifying the peak and nadirs of breaths using a custom MATLAB script. VT was calculated via a barometric method incorporating the flow rate, mouse weight and temperature, air temperature and amplitude of the metred breaths during calibration.^35^ VE was determined as the product of fR and VT. All data were normalized to baseline pre-stimulation values. For presentation as a time series, the breathing data were binned into 20 s epochs and averaged.

### Tissue processing

#### Intracardiac perfusion

Animals that succumbed to MES were prepared for transcardial perfusion immediately to preserve tissue quality. Surviving animals and the animals used for retrograde tracing were prepared for perfusion with an overdose of ketamine/xylazine (i.p., 50–75 and 5–7.5 mg/kg). The depth of anaesthesia was confirmed by toe pinch. An incision was made under the ribcage to expose the diaphragm, which was cut to reveal the heart. The right atrium was punctured to produce an area of outflow, and then a 25 GA needle attached to a variable flow mini-pump (3389; VWR International) was inserted into the left ventricle. Blood was flushed from circulation by perfusing chilled (4°C) 1X phosphate-buffered saline (PBS; 14190144; Gibco, Jenks, OH, USA) at ∼10 mL/min until the liver paled. 1X PBS was followed by chilled 4% paraformaldehyde (PFA; 101176-014; VWR International) until the neck and extremities stiffened. Heads were post-fixed in 4% PFA for 24 h at 4°C to facilitate observation of the implant site. The brains were then extracted and placed in a cryoprotection solution (30% sucrose in 1X PBS) for 72 h at 4°C. The tissue was embedded in Tissue-Tek optimum cutting temperature compound (VWR International) and stored at −80°C until it was sectioned on a cryostat (25 μm; CM1950; Leica Biosystems, Buffalo Grove, IL, USA).

#### Histology

Verification of genotype and implant location was confirmed via immunohistochemistry. The tissue was washed 4–6 times in 1X PBS prior to incubation with primary antibodies overnight. Primary and secondary antibodies were diluted with 1X PBS containing 0.25% Triton-X (A16046.AP; Thermofisher, Waltham, MA, USA), 2% normal donkey serum (017-000-121; Jackson Immunoresearch, West Grove, PA, USA) and 0.05% NaN_3_ (BDI#BDH7465-2; VWR International) to permeabilize tissue membranes, block non-specific binding and prevent bacterial contamination, respectively. After overnight exposure to primary antibodies, the tissue was then washed in 1X PBS 4–6 times before a 2 h exposure to secondary antibodies. Primary and secondary antibodies for each experiment. *TPH2-ChR2-YFP*/WT: Primary antibodies, mouse anti-TpOH (1:30 000; MAB5278; EMD Millipore, Burlington, MA, USA) and chicken anti-GFP (1:3000; A10262; Invitrogen, Carlsbad, CA, USA). Secondary antibodies, Cy3 donkey anti-mouse (1:500; 715165150; Jackson Immunoresearch) and Alexa 488 donkey anti-chicken (1:500; 703545155; Jackson Immunoresearch). *Pet1*-WT + Fluorogold: Primary antibody, rabbit anti-TPH2 (1:8000; NB100-74555; Novus Biologicals, Centennial, CO, USA). Secondary antibody, Cy3 donkey anti-rabbit (1:500; 715165152; Jackson Immunoresearch).

*In situ* hybridization was utilized to examine 5-HT_2A_ receptors within the BNST. RNA transcripts for 5-HT_2A_ (Mm-Htr2a-C2, 401291) and corticotrophin-releasing hormone (CRH; Mm-CRH-C1,316091) were fluorescently labelled via proprietary double-Z probes and the RNAscope fluorescent kit (Multiplex Fluorescent Reagent Kit v2; 323136; Advanced Cell Diagnostics Bio, Newark, CA, USA). Visualization of the probes was achieved via enzyme-mediated deposition of tyramine signal amplification–based fluorescent dyes (1:1500; Opal 520, FP1487001KT; Opal 570, FP1488001KT; Akoya Biosciences, Marlborough, MA, USA).

Fluorescent images were obtained via confocal microscopy and post-processed in OlyVIA (Olympus Life Science, Center Valley, PA, USA). Anatomic landmarks were identified with the aid of a mouse brain atlas.^36^ Mice with implants within the dorsomedial DRN were classified as DRD hits. Animals with implants in any other DRN subregion were classified as DRD miss and pooled for these experiments. Animals with implants that damaged >50% of the DRN, animals with implants that were too dorsal to affect the DRN, animals with large tears near the DRN and animals which experienced health issues following surgery were excluded from analysis.

#### c-Fos quantification

Optic fibres were implanted above the DRD in a separate cohort of *TPH2-ChR2-YFP* (*n* = 8) and WT (*n* = 3) animals to quantify c-Fos immunoreactivity. All animals underwent habituation to the toothless alligator clips and the recording chamber as previously described. However, these animals did not undergo MES. On the day following habituation, optogenetic stimulation (473 nm, 4 Hz, 10 mW, 5 ms) identical to the MES experiments was applied for 300 s. Animals were sacrificed 1.5 h after optogenetic stimulation to account for translation. Increased c-Fos in TPH2-ChR2 animals could support cell activation due to optogenetic light stimulation. Brain tissue was processed for IHC, as previously described. Primary antibodies: mouse anti-TpOH (1:30 000; MAB5278; EMD Millipore), chicken anti-GFP (1:3000; A10262; Invitrogen) and rabbit anti-cFos (1:1000; ABE457; EMD Millipore). Secondary antibodies: Cy5 donkey anti-mouse (1:500; 715175151; Jackson Immunoresearch), Alexa 488 donkey anti-chicken (1:500; 703545155; Jackson Immunoresearch) and Cy3 donkey anti-rabbit (1:500; 711165152; Jackson Immunoresearch). TPH2 expression was also quantified to ensure similar cell numbers existed in the *TPH2-ChR2-YFP* and WT sections used for quantification. One section was analysed per animal in a consistent 1.0 × 1.7 mm^2^ area surrounding the DRN.

#### Vaginal cytology

Estrus state was evaluated in female mice ≤4 h before MES induction using vaginal cytology. Vaginal secretions were collected by flushing the vaginal canal (∼120 μL) with room-temperature saline from a graduated transfer pipette (1 mL; 222NL; Thermo Fisher Scientific, Rockford, IL, USA) placed outside the vaginal opening.^37^ The saline was flushed 1 or 2 times to obtain a smear. The mice were categorized as being in proestrus, estrus, metestrus or diestrus based on the proportion of cornified and nucleated epithelial cells and leucocytes.^31^ We were not powered to detect differences between estrus cycle phases, but the distribution of estrus phases in the mice was: estrus (*n* = 12), metestrus (*n* = 5), diestrus (*n* = 11) and undetermined (*n* = 1).

#### Statistical analyses

Threshold for statistical significance was set at *P* < 0.05 for all comparisons. The normality of the data was assessed via a Shapiro–Wilk normality test. Independent two-tailed *t*-tests were utilized for parametric between-subjects comparisons for MES E/F ratio and seizure duration data. One-way ANOVAs were utilized for WT versus *TPH2-ChR2-YFP* comparisons in animals that survived versus died. Plethysmography data comparisons of frequency, tidal volume and minute ventilation averaged across the stimulation period were conducted using a Kruskal–Wallis non-parametric test with Dunn’s for multiple comparisons. An independent *t*-test with Welch’s correction for unequal standard deviations was utilized for analysis of c-Fos immunoreactivity data. Time series plethysmography data were assessed using a mixed-effects model with correction via Sidak’s multiple comparisons test. Mortality was assessed using Fisher’s exact test. Post hoc power analyses were conducted to confirm ≥0.80 β power for experiments (G*Power; Heinrich Heine University Düsseldorf). Graphpad Prism 7 software was utilized for visualizing results and statistics. Calculations to test post hoc power for the mortality data were designed using a dichotomous endpoint with two independent samples.^38^

## Results

### Optogenetic activation of DRN 5-HT neurons in pooled WT and *TPH2-ChR2-YFP* did not affect mortality following MES induction

**Figure 1:**
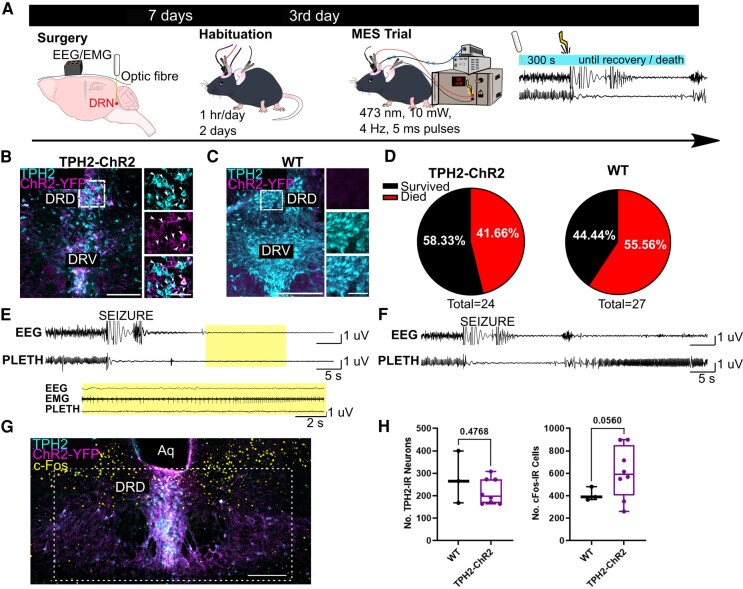
**Terminal apnoea preceded death in *TPH2-ChR2-YFP* which succumbed to MES.** (**A**) Schematic expression of the experimental timeline. (**B**) Immunostained coronal section, demonstrating ChR2-YFP expression, TPH2 expression and co-expression (arrows) within the DRN of a *TPH2-ChR2-YFP* (**B**) and WT (**C**) mouse. Box indicates location of insets (*top*, 5-HT cells; *middle*, ChR2-YFP; *bottom*, merge). Scale bars in B, 200 µm left, 20 µm right; Scale bars in C, 250 µm left, 50 µm right. (**D**) Mortality rate following optogenetic stimulation and MES in the pooled WT and *TPH2-ChR2-YFP* mice. *TPH2-ChR2-YFP*: *n* = 24, 10M, 14F; WT: *n* = 27, 12M, 15F. (**E**, **F**) Representative EEG and plethysmography traces (60 s) of *TPH2-ChR2-YFP* animals which either survived (**E**) or died (**F**) after optogenetic stimulation and undergoing MES. Inset in (**E**) demonstrates continued EKG activity during terminal apnoea in a mouse which died. Scale bars: horizontal, 5 s; vertical 1, 5, 1 μV. Error bars = SEM. (**G**) Coronal section demonstrating ChR2-YFP expression, TPH2 expression and c-Fos within the DRN of a *TPH2-ChR2-YFP* mouse following optogenetic stimulation of the DRD. Box indicates cell counting location. Scale bar = 200 µm. (**H**) Number of cells exhibiting TPH2 or c-Fos immunoreactivity (TPH2/c-Fos-IR) in *TPH2-ChR2-YFP* (*n* = 8) and WT mice (*n* = 3). *P* = 0.056; *t* = 2.201, d*F* = 8.776; Unpaired *t*-test with Welch’s correction.Dots = individual animals. Aq, aqueduct; DRD, dorsomedial dorsal raphe; DRV, dorsal raphe ventral; PLETH, plethysmography.

Mice (*TPH2-ChR2-YFP*: *n* = 24, 10M, 14F; WT: *n* = 27, 12M, 15F) were implanted with EEG/EMG electrodes and an optic fibre aimed at the DRN, as described above. On the day of trials, light stimulation was applied to the DRN for 300 s prior to a seizure-induced with a 50 mA transauricular current and persisted until first breath or death (Fig. 1A). *TPH2-ChR2-YFP* mice expressed ChR2-YFP in TPH2 containing (e.g. serotonergic) neurons (Fig. 1B), whereas no ChR2-YFP was expressed in the WT littermates (Fig. 1C). Survival rates in the *TPH2-ChR2-YFP* (survived: *n* = 14; 58.33%; died: *n* = 41.66%) and WT (survived: *n* = 11; 40.7%; died: *n* = 16; 59.2%) mice (Fig. 1D) were consistent with reported rates in the literature.^21,25^ This may be visualized by raw EEG and plethysmography traces from an animal that survived MES (Fig. 1E) versus died (Fig. 1F). As seen in prior MES studies, terminal apnoea preceded terminal asystole^22^ in the animals who succumbed to MES. These mice were not outfitted with EKG, but heartbeats were detectable on EMG after terminal apnoea in all cases (Fig. 1E). Optogenetic stimulation of the DRD in a separate cohort of animals resulted in a trend towards greater c-Fos immunoreactivity (*P* = 0.056; *t* = 2.201, d*F* = 8.776; unpaired *t*-test with Welch’s correction; Fig. 1G and H) in *TPH2-ChR2-YFP* animals (*n* = 8) compared with WT animals (*n* = 3).

### Optogenetic activation of DRN 5-HT neurons in pooled WT and *TPH2-ChR2-YFP* did not affect seizure severity or duration

**Figure 2:**
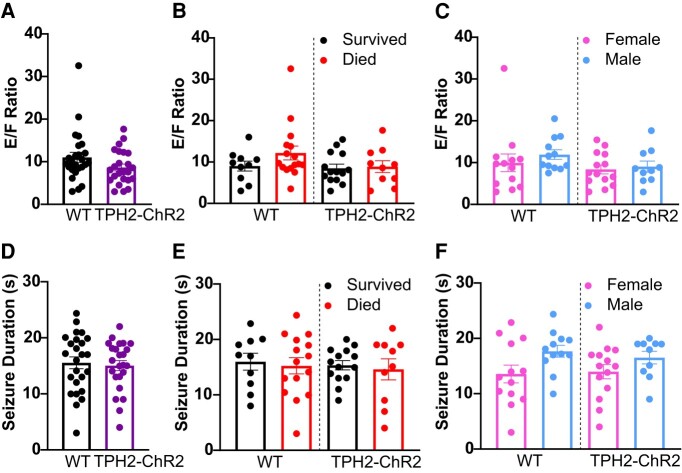
**Seizure severity and duration were similar between pooled WT and *TPH2-ChR2-YFP* mice.**  *TPH2-ChR2-YFP*: *n* = 24, 10M, 14F; WT: *n* = 25, 12M, 13F. *E*/*F* ratio (**A–C**) and seizure duration (**D–F**) between all WT (*n* = 25) and *TPH2-ChR2-YFP* mice (*n* = 24) (**A**, **D**), those that survived (WT: *n* = 10, 4M, 6F; *TPH2-ChR2-YFP*: *n* = 14, 5M, 9F) versus died from the MES procedure (WT: *n* = 15, 10M, 5F; *TPH2-ChR2-YFP*: *n* = 10, 5M, 5F) (**B**, **E**), and between the female and male mice of each genotype (**C**, **F**). Both WT and *TPH2-ChR2-YFP* mice received pre-ictal optogenetic stimulation. Dots = individual animals. Error bars = SEM. A–F: *P* ≥ 0.05; **A**: Mann–Whitney U test; **B**, **C**, **E**, **F**: one-way ANOVA; **D**: independent *t*-test.

Seizure severities, as assessed by the *E*/*F* ratios, were comparable between WT (11.03 ± 1.20; *n* = 25, 12M, 13F) and *TPH2-ChR2-YFP* mice (8.65 ± 0.811; *n* = 24, 10M, 14F; Mann–Whitney U test, *P* = 0.1089; Fig. 2A) and between mice that survived (WT: 9.02 ± 1.20; *n* = 10, 4M, 6F; *TPH2-ChR2-YFP*: 8.49 ± 0.98; *n* = 14, 5M, 9F) versus died (WT: 12.18 ± 1.68; *n* = 15, 10M, 5F; *TPH2-ChR2-YFP*: 8.88 ± 1.45; *n* = 10, 5M, 5F; one-way ANOVA, *P* = 0.1822, *F*(3, 46) = 1.690; Fig. 2B). *E*/*F* ratios were similar across sex and genotype (WT: males: females: 9.96 ± 2.11; *n* = 13; 11.92 ± 1.15; *n* = 12; *TPH2-ChR2-YFP*: females: 8.37 ± 1.05; *n* = 14; males: 9.04 ± 1.33; *n* = 10; one-way ANOVA, *P* = 0.369, *F*(3, 45) = 1.077; Fig. 2C). Seizure duration was not different between WT (15.54 ± 1.05 s; *n* = 25) and *TPH2-ChR2-YFP* mice (15.04 ± 0.90 s; *n* = 24; independent *t*-test, *P* = 0.7207, *t* = 0.3596, df = 47; Fig. 2D) or those which survived (WT: 15.98 ± 1.53 s; *n* = 10; *TPH2-ChR2-YFP*: 15.35 ± 1.33 s; *n* = 14) versus died [WT: 15.25 ± 1.46 s; *n* = 15; *TPH2-ChR2-YFP*: 14.61 ± 1.90 s; *n* = 10; one-way ANOVA, *P* = 0.9425, *F*(3, 45) = 0.1288; Fig. 2E]. Male and female mice experienced similar seizure durations [WT: 17.67 ± 1.07 s versus 15.65 ± 1.77 s; *n* = 12M, 12F; one-way ANOVA, *P* = 0.0951, *n* = 12F, 12M; WT, *F*(3, 45) = 2.253, Fig. 2F].

### Optogenetic activation of DRD 5-HT neurons reduced mortality without affecting seizure duration or severity

**Figure 3:**
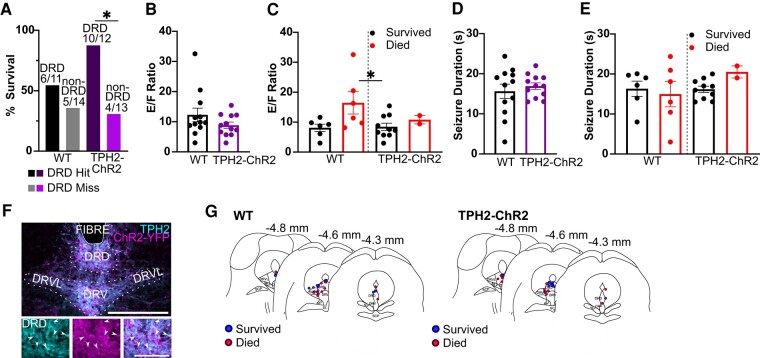
**Activation of DRD 5-HT neurons reduced MES mortality in *TPH2-ChR2-YFP* mice, but not WT littermates.** (**A**) Per cent survival of MES following optogenetic stimulation in male and female WT (survived: *n* = 6; 3M, 3F; died: *n* = 5; 2M, 3F) and *TPH2-ChR2-YFP* mice (survived: *n* = 10, 3M, 7F; died: *n* = 3, 1M, 2F) with optic fibres implanted within the DRD (*P = 0.0472, Fisher’s exact test). *E*/*F* ratio (**B**, **C**) and seizure duration (**D**, **E**) in WT and *TPH2-ChR2-YFP* with DRD implants (**B**, **D**) and between those that survived (WT: *n* = 6, 3M, 3F; *TPH2-ChR2-YFP*: *n* = 10, 7M, 3F) versus died from the MES procedure (WT: *n* = 5, 2M, 5F; *TPH2-ChR2-YFP*: *n* = 2, 1M, 1F) (**C**, **E**). (**F**) Immunostained coronal section depicting a DRD optic fibre implant. Box indicates location of insets (left, 5-HT cells; middle, ChR2-YFP; right, merge). Dashed lines denote raphe subdivisions. Scale bar = 500 µm, 200 µm. (**G**) Illustration of midbrain coronal sections demonstrating locations of all DRD hit implants in *TPH2-ChR2-YFP* and WT mice and whether the animal survived or died from the MES procedure. Dots = individual animals. Error bars = SEM. **B**–**E**: *P* = >0.05; **B**, **D**: independent *t*-test; **C**, **E**: one-way ANOVA. DRD, dorsomedial dorsal raphe; DRV, ventral dorsal raphe; DRVL, ventrolateral dorsal raphe; Fibre, optic fibre implant.

Pre-ictal activation of neurons within the DRD subdivision of the DRN reduced mortality in *TPH2-ChR2-YFP* mice (survived: *n* = 10, 3M, 7F; 76.9%; died: *n* = 3, 1M, 2F; 23.1%; *P* = 0.0472; Fisher’s exact test) compared with DRD hit WT littermates (survived: *n* = 6; 3M, 3F; 54%; died: *n* = 5; 2M, 3F; 46%; Fig. 3A). *E*/*F* ratio (WT hit: 12.27 ± 2.25; *n* = 12, 4M, 7F; *TPH2-ChR2-YFP* hit: 8.85 ± 1.03; *n* = 12, 4M, 8F; independent *t*-test, *P* = 0.1819, *t* = 1.378, df = 22; Fig. 3B) and seizure duration (WT: 15.42 ± 1.84 s; *TPH2-ChR2-YFP*: 16.91 ± 0.82 s; independent *t*-test, *P* = 0.5247, *t* = 0.6464, df = 22; Fig. 3D) were unaffected by pre-ictal activation of the DRD. There was no major differences in *E*/*F* ratio between animals that survived (WT: 8.10 ± 1.25; *n* = 6, 3M, 3F; *TPH2-ChR2-YFP*: 8.45 ± 1.19; *n* = 10, 7M, 3F) versus died [WT: 16.44 ± 3.72; *n* = 6, 2M, 4F; *TPH2-ChR2-YFP*: 10.82 ± 1.39; *n* = 2, 1M, 1F; one-way ANOVA, *P* = 0.0444, *F*(3, 20) = 3.225; Fig. 3C]. Seizure duration was unaffected between DRD hit animals that survived (WT: 15.42 ± 1.84; *n* = 6, 3M, 3F; *TPH2-ChR2-YFP*: 16.19 ± 0.77 s; *n* = 10, 7M, 3F) versus died (WT: 14.97 ± 3.16; *n* = 5, 2M, 5F; *TPH2-ChR2-YFP*: 20.52 ± 1.49 s; *n* = 2, 1M, 1F; Fig. 3E). In a small subsection of animals, *E*/*F* ratio and seizure duration were unable to be determined due to post-ictal body position. Mortality data were still utilized from these animals.

### Optogenetic activation of non-DRD sites within the DRN did not affect mortality or seizure severity and duration

**Figure 4:**
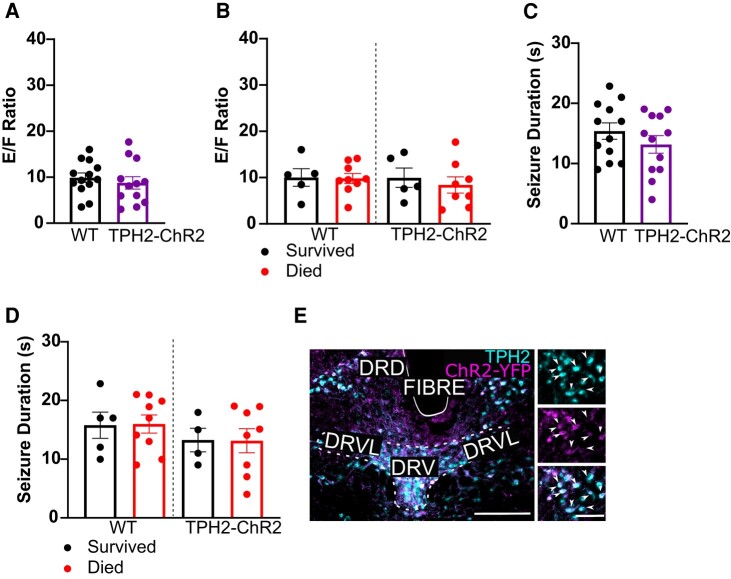
**Mortality rate was unaffected by activation of non-DRD 5-HT neurons in *TPH2-ChR2-YFP* mice and WT littermates.**  *E*/*F* ratio (**A**, **B**) and seizure duration (**C**, **D**) following optogenetic stimulation and MES in WT and *TPH2-ChR2-YFP* with non-DRD implants. WT: *n* = 15, 8M, 6F; *TPH2-ChR2-YFP*: *n* = 13, 5M, 8F. (**B**, **D**) and between those that survived (WT: *n* = 5, 2M, 3F; *TPH2-ChR2-YFP*: *n* = 5, 2M, 3F) versus died (WT: *n* = 9, 6M, 3F; *TPH2-ChR2-YFP*: *n* = 8, 3M, 5F) from the MES procedure (**B**, **D**). (**E**) Immunostained coronal section depicting an example non-DRD optic fibre implant. Box indicates location of insets (*top*, 5-HT cells; *middle*, ChR2-YFP; *bottom*, merge). Dashed lines denote raphe subdivisions. Scale bar = 250 µm, 100 µm. Dots = individual animals. Error bars = SEM. **A–D**: *P* ≥ 0.05; **A**, **C**: independent *t*-test; **B**, **D**: one-way ANOVA. Aq, aqueduct; DRD, dorsomedial dorsal raphe; DRV, ventral dorsal raphe; DRVL, ventrolateral dorsal raphe; Fibre, optic fibre implant.

Pre-ictal activation of DRN 5-HT neurons within animals whose implants were not within the DRD did not affect *E*/*F* ratio (WT: 11.77 ± 1.42; *n* = 15, 8M, 6F; *TPH2-ChR2-YFP*: 8.76 ± 1.36; *n* = 13, 5M, 8F; independent *t*-test, *P* = 0.5097, *t* = 0.669, df = 23; Fig. 4A), mortality (survived: *n* = 5; 33.3%; died: *n* = 10; 66.7%; Fig. 4B) or seizure duration (WT: 15.40 ± 1.35 s; *TPH2-ChR2-YFP*: 13.17 ± 1.46 s; independent *t*-test, *P* = 0.2764, *t* = 1.116, df = 22; Fig. 4C). *E*/*F* ratio and seizure duration were also consistent between DRD miss animals which survived (*E*/*F*: WT, 10.02 ± 1.91 versus *TPH2-ChR2-YFP*, 9.95 ± 2.08; Sz duration: WT, 15.78 ± 2.24 versus *TPH2-ChR2-YFP*, 13.25 ± 2.00 s) versus died (*E*/*F*: WT, 9.81 ± 1.23 versus *TPH2-ChR2-YFP*, 8.40 ± 2.23; Sz duration: WT, 15.36 ± 1.60 s versus *TPH2-ChR2-YFP*, 13.13 ± 2.04 s) from the MES procedure [one-way ANOVA, *P* = 0.8789, *F*(3, 22) = 0.2237; one-way ANOVA, *P* = 0.7097, *F*(3, 21) = 0.4652; Fig. 4B and E].

### Pre-ictal activation of DRD 5-HT neurons does not affect breathing

**Figure 5:**
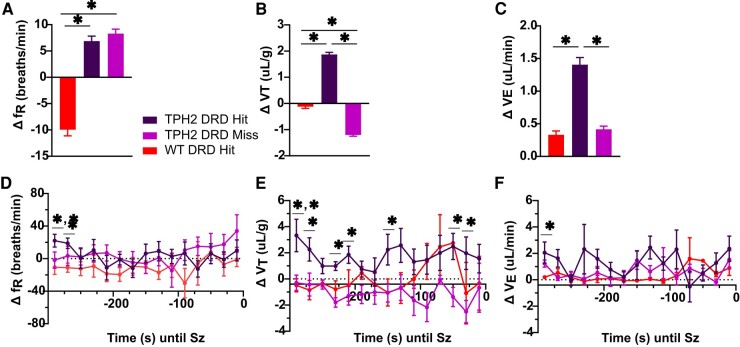
**Activation of DRD 5-HT neurons did not affect peri-ictal minute ventilation in *TPH2-ChR2-YFP* mice.** Change in average breathing frequency (breaths/min) (**A**), tidal volume (**B**) and minute ventilation (**C**) during optogenetic stimulation of *TPH2-ChR2-YFP* DRD hit (*n* = 7, 2M, 5F) and DRD miss mice (*n* = 6, 2M, 4F), and WT DRD hit mice (*n* = 5). Groups consisted of mixed sexes. Dots = individual animals. (*P < 0.0001, Kruskal–Wallis non-parametric test with Dunn’s for multiple comparisons. **A**: KW = 240.7, **B**: KW = 1350.0, **C**: KW = 58.31). Time series of breathing frequency (**D**), tidal volume (**E**) and minute ventilation (**F**) throughout the 300 s of optogenetic stimulation immediately prior to seizure induction (*P < 0.05, mixed-effects model with Sidak’s correction). Dots = group average over 20 s bins. All data were normalized to baseline pre-stimulation values. Error bars = SEM.

The average breathing frequency during optogenetic stimulation was unchanged between *TPH2-ChR2-YFP* mice with DRD hits (6.88 ± 0.94 br/min; *n* = 7, 2M, 5F) and misses (8.30 ± 0.86 br/min; *n* = 6, 2M, 4F), although WT mice with DRD hits exhibited lower average breathing frequency (−9.95 ± 1.172 br/min; *n* = 5, 5F; *P* < 0.0001; KW = 240.7; Kruskal–Wallis non-parametric test with Dunn’s for multiple comparisons; Fig. 5A) when compared with *TPH2-ChR2-YFP* DRD hits and misses. *n* increase in the average tidal volume was observed in *TPH2-ChR2-YFP* DRD hit mice (1.87 ± 0.07 μL/g; *P* < 0.05) compared with *TPH2-ChR2* DRD miss mice (−1.20 ± 0.05 μL/g; *P* < 0.0001) and WT mice with DRD hits (−0.124 ± 0.067 μL/g; *P* < 0.0001; KW = 1350.0; Kruskal–Wallis non-parametric test with Dunn’s for multiple comparisons; Fig. 5B). This contributed to a small overall change in average minute ventilation during optogenetic stimulation between *TPH2-ChR2-YFP* mice with DRD hits and misses (1.40 ± 0.11 versus DRD miss: 0.42 ± 0.05 μL/g/min; *P* = <0.0001; KW = 58.31; Kruskal–Wallis non-parametric test with Dunn’s for multiple comparisons; Fig. 5C). But, when the frequency, tidal volume and minute ventilation during optogenetic stimulation were presented as a time series, there were no consistent, clinically relevant differences in any respiratory parameters between *TPH2-ChR2-YFP* mice with DRD hits or misses or WT mice with DRD hits (Fig. 5D–F).

### Retrogradely labelled cells were identified in the DRD following fluorogold injections into the BNST and BLA

**Figure 6:**
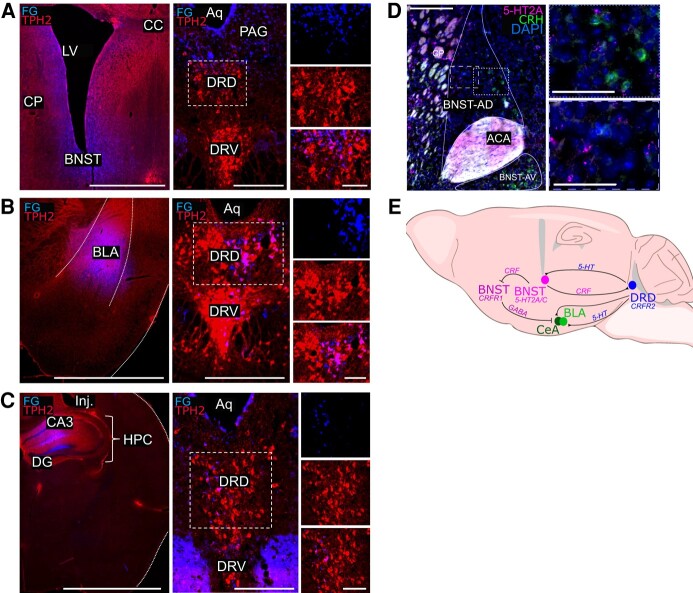
**Projections were identified from DRD to stress-modulating nuclei, such as the BNST and BLA, but not the HPC.** Immunostained coronal sections demonstrating TPH2 expression, fluorogold expression and co-expression within the DRD of a WT mouse following injections of FG into the BNST (**A**), BLA (**B**) and HPC (**C**). Box indicates location of insets (*top*, FG; *middle*, TPH2; *bottom*, merge). (**D**) Coronal sections from a mouse demonstrating 5-HT_2A_ and CRH RNA transcripts within the anterior-dorsal and anterior-ventral compartments of the BNST. (**E**) Sagittal section depicting potential DRD to BNST/Amygdala circuitry. ACA, anterior commissure; Aq, aqueduct; BLA, basolateral amygdala; BNST, bed nucleus of the stria terminalis; BNST-AD, anterior-dorsal BNST; BNST-AV, anterior-ventral BNST; DAPI, 4′,6-diamidino-2-phenylindole; DG, dentate gyrus; DRD, dorsomedial dorsal raphe; DRV, dorsal raphe ventral; CA3, cornus ammonis 3; CC, corpus callosum; CP, caudate putamen; CRF, corticotrophin releasing factor; CRFR1 or 2, corticotrophin-releasing factor receptor 1 or 2; GABA, gamma-aminobutyric acid; HPC, hippocampus; Inj, injection; LV, lateral ventricle; PAG, periaqueductal grey. Scale bars: **A** = 1 mm, 250 µm, 100 µm; **B** = 2 mm, 500 µm, 100 µm; **C** = 2.5 mm, 250 µm, 100 µm; **D** = 250 µm, 100 µm.

To assess potential downstream targets of the DRD, FG was injected into two sites suggested by the literature (e.g. BNST, BLA). FG was also injected into the HPC, a site reported to have sparse inputs from the DRD. Retrogradely labelled cells (blue) were identified within the DRD following injections into the BNST and BLA. Many 5-HT cells (red) displayed co-labeling with the retrogradely labelled cells from the BNST or BLA (Fig. 6A and B). In contrast, few retrogradely labelled cells were present in the DRD following an injection of FG into the HPC (Fig. 6C).

## Discussion

In this study, we observed that pre-ictal activation of 5-HT neurons within the DRD subnucleus of the DRN-reduced mortality from MES-induced seizures without affecting breathing (Figs 3 and 5). There was a trend towards greater c-Fos immunoreactivity in the *TPH2-ChR2-YFP* mice compared with the WT mice, suggesting that optogenetic light stimulation is activating cells within the DRN. However, c-Fos expression was observed in a myriad of locations other than the DRD (Fig. 1).

The seizure severities and durations observed in our mice were consistent with values published in prior studies.^21,23,39^ This suggests that the mice did not experience severe seizures that could have contributed to increased mortality rates in some groups. However, a trend towards an increase in seizure duration was observed in male WT mice compared with the female WT mice (Fig. 2). This could be due to the experimental manipulations because it is known that sex steroids can play a major role in seizure susceptibility to MES.^40,41^ However, the females were randomly assigned to groups, and estrus states were naturally, unevenly spread between groups. It is possible this finding is due to differences in estrus states in the female mice across groups. This difference in seizure duration did not translate towards increased survivability of WT mice in MES.

At least six major subdivisions of the DRN have been proposed based on cytoarchitecture: dorsal raphe rostral (DRNR), DRD, DRV, DRVL (‘lateral wings’), dorsal raphe intrafascicular (DRI) and dorsal raphe caudal (DRC).^26,42^ The DRN begins in the caudal midbrain as the DRNR and spreads in an anteroposterior axis along the cerebral aqueduct between the levels of the oculomotor nucleus, trochlear nucleus and abducens nucleus.^43^ Moving caudally, 5-HT cells are present in a compact zone ventromedially (DRD) and laterally (DRVL) to the cerebral aqueduct. A cell-sparse zone separates the DRD and DRNVL from the DRV and DRI at some levels, with the medial longitudinal fasciculus anatomically demarking the dorsal portion of the DRN from the ventral portion and the median raphe nucleus.^26,43^ These subdivisions vary in chemoarchitecture, gene expression^44^ and circuit connectivity.^42^

The DRD is serotonergic but also consists of nitrergic,^45^ glutamatergic,^46^ GABAergic^47^ and NADPH-expressing^48^ neurons. Several neuropeptides are also expressed in the DRD neurons, including corticotrophin-releasing factor (CRF),^49^ galanin^45^ and somatostatin^50^ in 5-HT neurons, neurokinin^51^ in glutamatergic neurons and vasoactive intestinal peptide in putatively non-5-HT neurons.^52^

A unique feature of the DRD is its role in stress and defensive behaviours.^53,54^ The bulk of research on this subnucleus focuses on its role in affective disorders; thus, we approached contemplation of a prospective circuit from this angle. The DRD expresses CRF,^49,55^ CRF receptor 1 (CRFR1) and CRF receptor 2 (CRFR2) in 5-HT neurons.^56^ CRF is a neuropeptide involved in anxiety, coping behaviours and activating the hypothalamic-pituitary axis in response to stress.^57,58^ Anterograde- and retrograde-tracing studies suggest DRD neurons may influence stress responses via reciprocal connections to structures, such as (but not limited to) the BNST,^49,59,60^ BLA^53^ and central amygdala (CeA).^60-62^

The BNST is a crucial node for integrating the emotional valence of stressors, coordinating appropriate stress responses and promoting arousal to stressors.^58,63^ The DRD projects to^59,60,64^ and receives projections from^54,65^ the oval BNST. This oval BNST contains CRF-expressing GABAergic neurons^66^ and facilitates anxiogenic behaviours by either inhibiting the dorsomedial BNST^67-69^ or by promoting arousal through excitatory CRF projections to regions, such as the lateral hypothalamus^70^ and periventricular nucleus of the hypothalamus.^70,71^

Interestingly, DRD projections to the oval BNST may be inhibitory or excitatory depending on prior stress exposure.^72^ Due to the high concentration of CRFR2, the DRD is likely anxiogenic and promotes stress responses.^56^ Periods of chronic stress can traffic CRFR2 to the plasma membrane and cause CRFR1 to undergo endocytosis.^55,73^ Generally, CRFR1 activation in DRD neurons reduces acute stress responses by inhibiting 5-HT_1A_-receptor expressing BNST neurons.^64,74^ However, CRFR2 activation is anxiogenic and promotes stress responses via activation of CRF-expressing BNST neurons via the 5-HT_2A/C_ receptors.^64,75^ Our preliminary staining also indicates the presence of the 5-HT_2A_ receptor on CRH containing neurons within the anterior-dorsal subcompartment of the BNST, but not in the anterior-ventral region (Fig. 6D). CRH and CRF are synonymous molecules, thus staining for CRH RNA transcripts indicates cells expressing CRF. It is possible that activation of this unique population of CRFR2-expressing 5-HT cells within the DRD-reduced mortality of MES seizures by promoting arousal before, during and immediately after the seizure (Fig. 6E).

The DRD is also involved in promoting anxiogenic and defensive responses via connections to the BLA^53^ (Fig. 6B). Emotionally arousing stimuli promote the release of stress hormones which can enhance later memory consolidation by acting on adrenoreceptors within the BLA.^76^ Like the DRD, the BLA is an extrahypothalamic source of CRF-containing neurons and many neurons express CRF1 and 2.^56^ CRF tone rises in the amygdala following exposure to stressors (e.g. acute restraint and ethanol withdrawal) in rats.^77^ Conversely, rats display anxiety behaviours (spent less time in the open arm of an elevated plus maze) after injections of a CRF1 and 2 agonists (UCN) into the BLA over 5 days.^78^

DRD 5-HT neurons may also modulate the BLA and subsequent stress responses. Inhibitory avoidance in a T-maze is facilitated following intra-DRD injections of 5-HT_1A_ antagonist, whereas a 5-HT_1A_ agonist reduces avoidance performance.^79^ Facilitation of inhibitory avoidance by the 5-HT_1A_ antagonist is blocked by the injection of a 5-HT_2C_ antagonist into the BLA.^53^ Therefore, DRD 5-HT neurons may act on 5-HT_2C_ receptors in the BLA to facilitate anxiogenic behaviour. Similarly, 5-HT levels in the BLA are augmented during social exploration in animals primed by uncontrollable tail shocks.^80^ This increase was blocked by intra-BLA injections of a 5-HT_2C_ antagonist.^80^ DRN 5-HT neurons expressing CRF1 or 2 may also be involved in a DRD to BLA circuit, as intra-DRN injections of UCN facilitate 5-HT efflux into the BLA.^81^ Thus, the DRD may facilitate anxiogenic responses through CRF or 5-HT containing projections to the BLA.

These pathways modulating stress responses may be relevant to SUDEP, as stress and epilepsy may be bidirectionally related.^82^ Patients across multiple epilepsy syndromes self-report that stress, sleep deprivation and fatigue exacerbate their seizures.^83,84^ Seizures themselves are stressful events and, thus, may contribute to stress-induced neural plasticity and changes to the hypothalamic-pituitary axis that could predispose one to future seizures.^85^ However, stress is a critical adaptive mechanism that allows us to arouse to and react appropriately to stimuli.^86^ Under normal circumstances, a sudden event causes neural stress networks to activate the hypothalamus, which in turn releases neurohormones into general circulation that stimulate the adrenal cortex to secrete epinephrine and norepinephrine.^86^ Subsequently, fight-or-flight physiological effects, such as sweating, dilated bronchial tubules and increased heart rate, can occur.^87^ Here, it is unlikely activation of the DRD-reduced seizure-induced mortality through the fight-or-flight response. Robust changes in breathing were not observed during the optogenetic stimulation between *TPH2-ChR2* mice with DRD hits and misses and WT mice with DRD hits (Fig. 5). When breathing frequency was averaged across the stimulation period, the WT mice with DRD hits exhibited lower breathing frequency compared with the *TPH2-ChR2* mice with DRD hits or misses. This could be attributed to reduced arousal in the WT mice, as they do not express ChR2. It could be that increased arousal through activation of the stress neural circuitry downstream the DRD hastened post-ictal recovery and reduced mortality.

Another possibility is that activation of DRD 5-HT neurons diminished neuronal synchrony and seizure propagation along amygdala pathways maintaining respiration. Terminal apnoea is the primary cause of death in the MES model,^21,22^ which is recapitulated in this study. This is likely due to a combination of central apnoea and tonic contraction of the diaphragm, although the latter has not been empirically shown. Studies from patients with epilepsy demonstrate that seizure propagation or stimulation of the amygdala can elicit apnoea.^28,88^ Similarly, CeA lesions in DBA/1 mice reduce the incidence of seizure-induced respiratory arrest (S-IRA) following audiogenic seizures.^29^

The DRD forms a feedback loop with the BNST, BLA and CeA, whereby a stressor’s valence is communicated by the CeA and modulates BNST activity.^59^ Then, the BNST forms a bidirectional feedback loop with the DRD,^68,89^ which then projects directly to the CeA^65^ or the BLA^53^ to alter amygdala output. Therefore, while activation of DRD 5-HT neurons did not alter breathing in this study, driving neuronal activity in the DRD before, during and after the seizure may have prevented seizure-induced disruption of brainstem respiratory centers. This is consistent with previous observations that ascending projections from the DRN are involved with arousal to stimuli such as CO_2_,^90^ whereas the caudal medullary raphe nuclei directly influence breathing via projections to brainstem nuclei.^21,33^

A recent study also explored the role of the BNST in S-IRA in DBA/1 mice.^30^ The authors detected a decrease in S-IRA following an injection of viral-mediated tetanus neurotoxin into the dorsal BNST. Tetanus toxin was expressed in both the dorsal and ventral portions of the BNST. Therefore, the exact site within the BNST mediating the reduction in S-IRA may be unclear. In the current experiments, cell-specific opsin expression and the anatomical location of the DRD (surrounded dorsally by the cerebral aqueduct) may have facilitated more precise activation of BNST inputs. Regardless, it is evident that altering firing in the BNST can affect the excitatory/inhibitory balance in downstream structures and is an important observation for epilepsy research.

To conclude, we identified a subset of DRN neurons critical for survival from a seizure. This is similar to an additional study where pre-ictal optogenetic stimulation of the DRN reduced S-IRA following acoustic stimulation in primed DBA/1 mice.^91^ Although we achieved a similar decrease in mortality/near SUDEP events, the laser parameters utilized by the authors are different (9 mW, 20 ms pulses, 20 Hz, over 15 min). This provides some additional support to the relevance of subcortical serotonergic arousal networks in preventing SUDEP. DRN connectivity with stress/arousal pathways, such as the BNST, may be salient targets in the prevention of seizure-related mortality. Further work will perform a similar experiment and target 5-HT fibres within the BNST and BLA with optogenetic stimulation. Ultimately, the goal of future experiments will be to dissect DRD circuitry and other potential targets, to determine which receptors and downstream structures are sufficient and necessary to reduce seizure-induced mortality.

## Data Availability

Raw data were collected at the University of Iowa. All data, protocols and scripts are available upon reasonable request to the corresponding author.
